# Evaluating the Use of Blood Cultures in the Management of Children Hospitalized for Community-Acquired Pneumonia

**DOI:** 10.1371/journal.pone.0117462

**Published:** 2015-02-06

**Authors:** Russell J. McCulloh, Michael P. Koster, Dwight E. Yin, Tiffany L. Milner, Shawn L. Ralston, Vanessa L. Hill, Brian K. Alverson, Eric A. Biondi

**Affiliations:** 1 Department of Pediatrics, Children’s Mercy Hospital, University of Missouri-Kansas City, Kansas City, MO, United States of America; 2 Department of Pediatrics, Rhode Island Hospital, Providence, RI, United States of America; 3 Department of Pediatrics, Dartmouth-Hitchcock Medical Center, Lebanon, NH, United States of America; 4 Department of Pediatrics, Children’s Hospital of San Antonio, San Antonio, TX, United States of America; 5 Department of Pediatrics, Rochester Medical Center, Rochester, NY, United States of America; University of Louisville, UNITED STATES

## Abstract

**Background:**

Blood cultures are often recommended for the evaluation of community-acquired pneumonia (CAP). However, institutions vary in their use of blood cultures, and blood cultures have unclear utility in CAP management in hospitalized children.

**Objective:**

To identify clinical factors associated with obtaining blood cultures in children hospitalized with CAP, and to estimate the association between blood culture obtainment and hospital length of stay (LOS).

**Methods:**

We performed a multicenter retrospective cohort study of children admitted with a diagnosis of CAP to any of four pediatric hospitals in the United States from January 1, 2011-December 31, 2012. Demographics, medical history, diagnostic testing, and clinical outcomes were abstracted via manual chart review. Multivariable logistic regression evaluated patient and clinical factors for associations with obtaining blood cultures. Propensity score-matched Kaplan-Meier analysis compared patients with and without blood cultures for hospital LOS.

**Results:**

Six hundred fourteen charts met inclusion criteria; 390 children had blood cultures obtained. Of children with blood cultures, six (1.5%) were positive for a pathogen and nine (2.3%) grew a contaminant. Factors associated with blood culture obtainment included presenting with symptoms of systemic inflammatory response syndrome (OR 1.78, 95% CI 1.10–2.89), receiving intravenous hydration (OR 3.94, 95% CI 3.22–4.83), receiving antibiotics before admission (OR 1.49, 95% CI 1.17–1.89), hospital admission from the ED (OR 1.65, 95% CI 1.05–2.60), and having health insurance (OR 0.42, 95% CI 0.30–0.60). In propensity score-matched analysis, patients with blood cultures had median 0.8 days longer LOS (2.0 vs 1.2 days, *P* < .0001) without increased odds of readmission (OR 0.94, 95% CI 0.45–1.97) or death (*P* = .25).

**Conclusions:**

Obtaining blood cultures in children hospitalized with CAP rarely identifies a causative pathogen and is associated with increased LOS. Our results highlight the need to refine the role of obtaining blood cultures in children hospitalized with CAP.

## Introduction

Pediatric community-acquired pneumonia (CAP) is a common reason for hospitalization, and CAP is associated with substantial healthcare resource use and costs to patients and families.[1] Previous studies have documented extensive variation in the management of CAP in the emergency department[2] and in the inpatient setting.[3] In 2011, national guidelines were developed as a means of optimizing healthcare resource use and clinical outcomes. These guidelines recommended specific measures to standardize the evaluation and treatment of CAP in children.[4] In particular, these guidelines recommend that “[b]lood cultures should be obtained in children requiring hospitalization for presumed bacterial CAP that is moderate to severe, particularly those with complicated pneumonia.” However, the authors noted that this strong recommendation was based on low-quality evidence, and criteria for obtaining blood cultures are often not followed.[5]

Previous studies have identified a low prevalence of bacteremia among children evaluated in the emergency department for CAP. A review of blood cultures obtained from children seen at 35 emergency departments found an overall prevalence of bacteremia of only 2.1% and a contamination prevalence of 1.0%.[6] Bacteremia prevalence is higher among children hospitalized for CAP, particularly if pneumonia is complicated by pleural effusion.[7] However, the overall prevalence of bacteremia in this population remains only 7%, with most cases among children undergoing pleural drainage or with other distal sites of infection (e.g., osteomyelitis).[7]

Given the national benchmark of blood culture contamination at 2–3%,[8–10] false-positive blood cultures pose a significant potential concern for pediatricians managing pediatric CAP in the inpatient setting. A previous study of the impact of contaminated blood cultures for hospitalized adults found that contaminated blood cultures resulted in an increased length of stay (LOS) of 4.5 days and significant increases in health care costs.[11] Thus, identifying the potential impact of obtaining blood cultures on the clinical course of children hospitalized for CAP is a critical need. The primary objectives of this study were (1) to identify factors associated with a medical provider’s decision to obtain blood cultures in children hospitalized with CAP and (2) to estimate the association of blood culture obtainment and hospital LOS. Secondary objectives were to assess any associations of obtaining blood cultures with other diagnostic evaluations, antibiotic prescription patterns, and clinical outcomes.

## Methods

### Data Source

We performed a retrospective cohort study of health care provider practices for pediatric CAP in four regionally distinct hospitals. This study was approved by the IRBs associated with each site, including the Committee for the Protection of Human Subjects (Children’s Hospital at Dartmouth, Lebanon, NH), the Lifespan Institutional Review Board (Hasbro Children’s Hospital, Providence, RI), the Research Subjects Review Board at Rochester Medical Center (Golisano Children’s Hospital at Rochester Medical Center, Rochester, NY), and the University of Texas Health Science Center Institutional Review Board (Children’s Hospital of San Antonio (San Antonio, TX). Written informed consent for obtainment of patient information was waived by all institutional IRBs under the condition that patient data would be de-identified. Data collected were de-identified/anonymized at each respective study site prior to aggregation and analysis. All data were extracted from existing medical records. Eligible participants included all otherwise healthy children aged 3 months to 18 years initially admitted to a general inpatient unit for CAP between January 1, 2011 and December 31, 2012, identified by admitting or discharge diagnosis of pneumonia by international classification of disease, ninth edition (ICD-9) codes 480–488.1. Exclusion criteria included direct admission to the PICU or the presence of ICD-9 codes indicating medical complexity, including chronic neuromuscular, cardiovascular, respiratory, renal, gastrointestinal, hematologic, immunologic, metabolic, congenital/genetic, or malignancy as defined elsewhere.[12] Patients initially admitted to an outside hospital or emergency department did not have available temporal data on blood culture obtainment, antimicrobial prescriptions, or total LOS. To avoid immortal-time bias,[13] these patients were also excluded from analysis.

### Data Collected

Reviewers manually collected: (1) demographic data; (2) diagnostic evaluation data; (3) antimicrobials administered; (4) therapeutic interventions; and (5) clinical outcomes including LOS, intensive care unit transfer, readmission within 14 days of discharge, and death during hospitalization. Pneumococcal and *Haemophilus influenzae* type b (Hib) vaccination status was also assessed, based on documentation within available medical records. Patients were considered fully immunized for these pathogens when: (1) vaccine records were available for review and were up to date; (2) parents or the care team reported immunizations being up to date and immunization records were unavailable; or (3) documentation of incomplete immunization status was absent. Patients were determined to meet criteria for systemic inflammatory response syndrome (SIRS) based on review of vital signs in the emergency department and hospital admission using international consensus guidelines criteria.[14] LOS was calculated using admission and discharge dates and times. Blood culture results were determined false-positive if any of the following criteria were met: 1) growth of an organism considered to be unlikely a pathogen in otherwise healthy children, including *Bacillus* non-anthracis spp. and *Corynebacterium* spp.; 2) growth of a single isolate of a typically non-pathogenic organism in otherwise healthy children including coagulase-negative staphylococci; 3) growth of multiple bacterial isolates of common contaminant skin flora such as coagulase-negative staphylococci with different resistance patterns; or 4) growth of a likely contaminating organism in a single blood culture at >48 hours. Multiple positive cultures of apparently identical (i.e. same resistance pattern) coagulase-negative staphylococci were to be considered true positives.

### Statistical Analysis

Univariable categorical analyses were performed using χ^2^ statistics or Fisher’s exact test, as appropriate. Continuous variables were analyzed using linear regression. The linearity of trends was assessed using cubic B-splines, and optimal coding of continuous variables were determined using Akaike information criterion and Bayesian information Criterion.

Models used subject-matter knowledge from published literature and *a priori* clinical assumptions, analyzed by directed acyclic graphs (DAG), to guide statistical modeling assumptions.[15,16] The DAG analysis served as the basis for constructing propensity scores. For the purposes of this study, “treated” patients were defined as those having blood cultures drawn; patients not undergoing blood cultures were “controls”. Controls were matched to treated in a 1:1 ratio using random sampling with replacement. To minimize bias, we restricted the caliper to 0.1 to ensure controls had similar probabilities of receiving treatment. We restricted the matched analyses to include only those patients with common support (positivity assumption)[17], which implies that the patient must have at least one control within their caliper to be included. Due to a larger sample of matched participants without blood cultures, controls were matched with replacement, resulting in a “pseudo-population” with oversampling of controls. The final sample of this pseudo-population control group was reweighted to restore the original control group sample size.

Potential predictors of blood culture obtainment were analyzed using multivariable logistic regression with generalized estimating equations, clustered by study site. Length of stay was compared using propensity score-matched Kaplan-Meier survival analysis. Patients were right-censored at death. As prolonged hospitalizations were less likely to be related to initial blood culture obtainment, we provided greater weight to earlier discharges by using the Peto-Peto-Prentice test statistic. This length of stay analysis was repeated after excluding patients with a pathogenic blood culture to estimate the hypothetical impact of eliminating all unnecessary (negative or contaminant) blood cultures. Secondary analyses assessed the relationship of obtaining blood cultures with other diagnostic evaluations, antimicrobial prescription practices, and clinical outcomes (intensive care unit [ICU] stay, hospital readmission, or death) using propensity score-matched logistic regression with generalized estimating equations, clustered by study site. If odds ratios were unable to be estimated due to rare events, we used propensity score-matched Fisher’s exact test. Statistical significance was defined as a two-sided *P* < .05. All analyses were performed using Stata SE, version 13.1 (STATACorp, College Station, TX), with the PSMATCH2 module used for propensity scoring.

## Results

### Eligibility and Baseline Factors

During the study period, a total number of 838 children met inclusion criteria. Of these patients, 75 were excluded for presence of a complex chronic condition, 148 were excluded for being admitted from a referring hospital or emergency department, and one patient was excluded for missing admission and discharge data, leaving a total of 614 patients who qualified for analysis. Patient demographics and baseline factors at admission are summarized in Table 1.

**Table 1:** Characteristics of children hospitalized for CAP, based on obtainment of blood cultures (n = 614).

Variable	Culture (N = 390)	No culture (N = 224)	Matched culture population (n = 386)	Matched no culture pseudo-population (n = 386)
Demographics/presentation				
Age (yrs, mean [SD])	5.2 (4.8)	5.1 (4.3)	5.1 (4.8)	4.9 (4.3)
Female gender	181 (46.3%)	109 (48.7%)	179 (46.4%)	201 (52.1%)
Preceding illness (days)	5.6 (4.7)	5.4 (5.5)	5.5 (4.8)	6.4 (7.6)
Received antibiotics pre-admit	155 (39.6%)	78 (34.8%)	150 (38.9%)	148 (38.3%)
Met SIRS criteria at presentation†	331 (84.7%)	179 (79.9%)	326 (84.5%)	318 (82.4%)
Received supplemental oxygen at presentation	205 (52.4%)	122 (54.5%)	201 (52.1%)	193 (50.0%)
Chest X-ray performed	376 (96.2%)	219 (97.8%)	371 (96.1%)	371 (96.1%)
Pleural effusion identified	219 (56.0%)	137 (61.2%)	219 (56.7%)	242 (62.7%)
Received intravenous hydration	346 (89.6%)	120 (82.2%)	346 (89.6%)	350 (90.7%)
Vaccinated for Hib, S. pneumo	372 (95.1%)	220 (98.2%)	368 (95.3%)	379 (98.2%)

†As defined in Goldstein, et. al., 2005;

Hib = *Haemophilus influenzae*, *type b*.

Three hundred ninety children had blood cultures obtained. Six (1.5%) children had blood cultures positive for a pathogen (5 patients with *Streptococcus pneumoniae*, 1 with non-typeable *Haemophilus influenzae*), and nine (2.3%) grew a contaminant (6 patients with coagulase-negative staphylococci, 1 patient with non-hemolytic streptococci, and 2 patients with *Bacillus* species, non-anthracis). Two patients with pneumococcal bacteremia were treated with narrow-spectrum antibiotics; the remainder of patients with bacteremia received broad-spectrum cephalosporin therapy at hospital admission and discharge. More than a third of patients received at least one dose of antibiotics either at an ambulatory clinic or in the ED prior to admission (39.6% with blood cultures, 34.8% without blood cultures). Children were generally identified as “up to date” for pneumococcal and Hib vaccination, and most children presented to the hospital with SIRS criteria. Clinical and patient factors included in the propensity score are listed in Table 2. No factor differed significantly between populations with and without blood cultures in propensity score-matched analysis (Table 1), suggesting that the matching procedure was successful.

**Table 2:** Factors evaluated for association with obtainment of blood cultures.

Factor	Unadjusted OR (95% CI)	Adjusted OR (95% CI)
Patient age (in years)	1.01 (0.99–1.02)	0.98 (0.96–1.01)
Female gender	0.90 (0.81–1.01)	0.88 (0.73–1.05)
Met SIRS criteria at presentation†	1.38 (0.74–2.59)	1.78 (1.10–2.89)
Received supplemental oxygen at presentation	0.92 (0.80–1.05)	0.80 (0.63–1.02)
Duration of preceding illness (in days)	1.00 (0.97–1.05)	0.99 (0.96–1.02)
Pleural effusion identified	0.81 (0.57–1.16)	0.79 (0.59–1.06)
Received antibiotics pre-admit	1.22 (0.95–1.57)	1.49 (1.17–1.89)
Received intravenous hydration	3.50 (3.23–3.79)	3.94 (3.22–4.83)
Insured	0.39 (0.33–0.46)	0.42 (0.30–0.60)
Admitted from ED	1.77 (1.12–2.79)	1.65 (1.05–2.60)

†As defined in Goldstein, et. al., 2005.

### Factors associated with blood culture obtainment

Table 2 describes the magnitude of association of selected criteria on the decision to obtain blood cultures. Factors associated with an increased odds ratio (OR) for blood culture obtainment included the presence of SIRS criteria at presentation (OR 1.78, 95% CI 1.10–2.89), receiving intravenous hydration (OR 3.94, 05% CI 3.22–4.83), receipt of antibiotics prior to admission (OR 1.49, 95% CI 1.17–1.89), and hospital admission from the ED (OR 1.65, 95% CI 1.05–2.60). Patients with insurance had fewer blood cultures drawn than patients with no health insurance (OR 0.42, 95% CI 0.30–0.60).

### Management of patients hospitalized with CAP

Table 3 summarizes diagnostic and treatment practices used in children hospitalized with CAP based on whether blood cultures were obtained. In propensity score-matched analysis, children from whom blood cultures were obtained had a greater prevalence of chest tube placement (4.9% vs. 0.0%, *P* < .0001) and ancillary diagnostic testing. Although receipt of antibiotics was similar between study groups, third- and fourth-generation cephalosporin use was more prevalent among children undergoing blood cultures. Children not undergoing blood cultures more often received narrow-spectrum antibiotic therapy.

**Table 3:** Management of children hospitalized for CAP, based on obtainment of blood cultures (n = 614).

Variable	Culture (N = 390)	No culture (N = 224)	Unmatched OR (95% CI)	Matched culture pseudo-population (n = 386)	Matched no culture pseudo-population (n = 386)	Matched OR (95% CI)
Additional evaluation/treatments						
CT scan	12 (3.1%)	1 (0.5%)	*	12 (3.1%)	0 (0.0%)	*
Chest Tube	19 (4.9%)	1 (0.5%)	11.4 (2.59–50.3)	19 (4.9%)	0 (0.0%)	*
CBC obtained	369 (94.4%)	116 (51.8%)	15.6 (4.94–49.1)	364 (94.3%)	223 (57.8%)	12.09 (4.63–31.57)
Serum chemistries	304 (77.8%)	94 (42.0%)	4.82 (2.89–8.03)	299 (77.5%)	198 (51.3%)	3.26 (1.89–5.65)
Urinalysis obtained	117 (29.9%)	28 (12.5%)	3.0 (1.90–4.73)	117 (30.3%)	59 (15.3%)	2.41 (1.72–3.37)
Urine culture obtained	145 (37.1%)	26 (11.6%)	4.51 (3.08–6.59)	143 (37.1%)	78 (20.2%)	2.32 (1.60–3.38)
Sputum culture done	114 (29.2%)	20 (8.9%)	4.21 (2.72–6.51)	112 (29.02%)	62 (16.1%)	2.13 (1.21–3.78)
Respiratory viral testing	95 (24.3%)	38 (17.0%)	1.58 (0.99–2.52)	95 (24.6%)	60 (15.5%)	1.77 (1.32–2.38)
Viral testing positive	53/95 (55.8%)	25/38 (65.8%)	0.66 (0.24–1.81)	53/95 (55.8%)	28/60 (46.7%)	1.44 (0.43–4.86)
Received abx on admit	348 (89.2%)	200 (89.3%)	0.99 (0.92–1.07)	344 (89.1%)	351 (90.9%)	0.82 (0.53–1.26)
Received 3^rd^/4^th^ gen cephalosporin	176 (50.6%)	54 (27.0%)	2.77 (2.11–3.61)	173 (50.3%)	108 (30.8%)	2.28 (1.3–4.0)
Received amox/amp at admission	95 (27.3%)	88 (44.0%)	0.48 (0.35–0.65)	94 (27.3%)	148 (42.2%)	0.51 (0.35–0.76)
Clinical outcomes						
Admitted to the ICU	52 (13.3%)	17 (7.6%)	1.87 (1.02–3.43)	52 (13.5%)	37 (9.6%)	1.47 (0.75–2.88)
Readmission w/in 14d	18 (4.6%)	8 (3.6%)	1.31 (.56–3.06)	17 (4.4%)	18 (4.7%)	0.94 (0.45–1.97)
Death	3 (0.8%)	0 (0.0%)	*	3 (0.8%)	0 (0.0%)	*

CBC = Complete blood count

* OR unable to be estimated due to rare events.

Unadjusted and adjusted Fisher’s exact p-values 0.04 and <0.0001, respectively; Fisher’s exact<0.0001. Unadjusted and adjusted fisher’s exact p-values are 0.56 and 0.25, respectively; Hib = *Haemiphilus influenzae*, *type b*; LOS = length of stay; abx = antibiotics.

### Clinical outcomes

**Fig 1:**
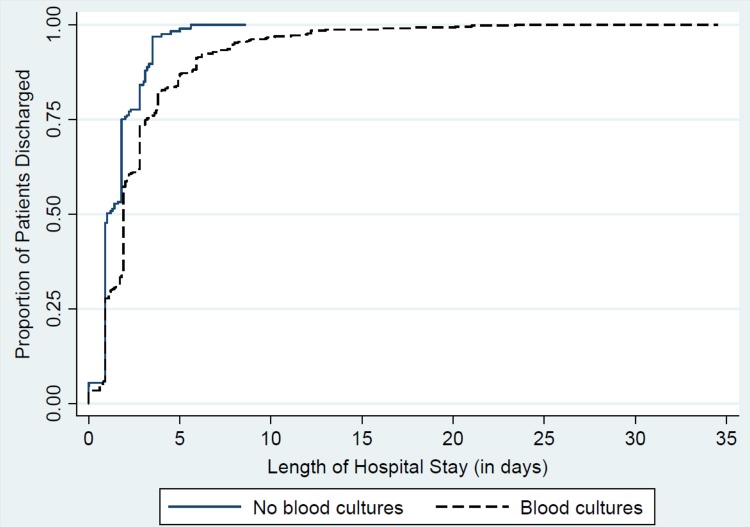
Kaplan-Meier survival curve for time to discharge for children hospitalized with CAP based on blood culture obtainment.

Times to discharge of patients undergoing blood cultures and not undergoing blood cultures are compared in Fig. 1. These results represent a difference in median time to discharge of 0.8 days (2.0 vs 1.2 days, *P <* .0001). An additional analysis excluding positive blood cultures (n = 6) found similar results (data not shown). In propensity score-matched analysis, patients with blood cultures had marginally higher odds of ICU stay (OR 1.47, 95% CI 0.75–2.88), but no significant difference in odds of re-hospitalization (OR 0.94, 95% CI 0.45–1.97; Table 3). There was also no association between obtainment of blood cultures and prevalence of patient deaths in unmatched (*P* = .56) or matched analysis (*P* = .25; Table 3). No re-hospitalizations were due to bacteremia or sepsis.

## Discussion

This multi-center, retrospective analysis of children hospitalized with CAP identified several factors associated with the decision to obtain blood cultures. We found that the presence of criteria consistent with SIRS, receipt of antibiotics prior to hospital admission, and receipt of intravenous hydration were all factors associated with an increased OR for blood culture obtainment. These findings are consistent with the IDSA recommendation for obtaining blood cultures in moderate to severe CAP, as they suggest illness which might be considered severe or unresponsive to outpatient therapy. Admission to the hospital from the ED was also associated with increased OR for blood culture obtainment, which is consistent with findings from previous studies of practice patterns for CAP management in the ED.[2,18] Having health insurance was associated with a lower OR for blood culture obtainment. Previous studies have described decreased resource use among children with public or no health insurance as compared to patients with private insurance.[19] These previous findings make the reduced OR we identified potentially somewhat surprising, as our cohort included a majority of children who had private insurance (60%).

Our study also quantified the potential magnitude of the effect that obtainment of blood cultures may have had on time to discharge for children hospitalized with CAP. We identified a median increase in time to discharge of 0.8 days for children undergoing blood cultures. We hypothesize that the median LOS of 2 days for patients undergoing blood cultures may reflect clinicians’ decision to await negative blood culture results at 48 hours. Our findings are consistent with recently-published studies in children hospitalized with CAP on the potential impact of blood culture testing on LOS.[20] Unlike the study published by Leyenaar, et al., our analyses were able to account for specific blood culture results, which included a contamination prevalence of 2.3% and detection of a pathogen in only 1.5%. These microbiological data allowed us to more precisely determine the potential benefit of a more targeted blood culture approach on LOS. Given the non-specific nature of the IDSA guidelines in regards to blood culture testing,[4] our results support more refined criteria for blood culture testing in children hospitalized for CAP.[21]

The decision to obtain blood cultures occurs concurrently with several other important decisions regarding the management of CAP. These decisions include use of other diagnostic testing modalities, choice and route of antimicrobial therapy, and even whether a patient should be hospitalized. Previous studies have found that blood culture obtainment has been associated with increased use of other ancillary testing and an increased risk of hospitalization.[2] The results of our study support these findings. In unmatched analysis, patients undergoing blood cultures had higher prevalence of nearly every diagnostic testing modality. One could argue that patients having blood cultures obtained are more ill at presentation, prompting health care providers to order blood cultures. However, after matching on propensity scores based on patient and clinical factors, the differences in ancillary testing persisted. ICU admission—a surrogate for illness severity that was intentionally excluded from our propensity score—was only marginally higher in children with blood cultures after propensity score matching, suggesting that illness severity was reasonably well-controlled in our analysis. Additionally, we found that blood culture obtainment directly correlated with use of third- and fourth-generation cephalosporins and inversely correlated with use of narrow-spectrum therapies. When taken together, these observations suggest that blood culture testing was more related to practice patterns than to clinical illness. Indeed, children with and without blood cultures had similar odds of death and readmission, suggesting that different practice patterns did not result in different patient outcomes. Thus, reducing unnecessary blood cultures may need to be part of a larger strategy to optimize overall diagnostic and antimicrobial use in pediatric CAP.[22,23]

Our study has limitations. First, the marginally higher frequency of ICU admissions in children with blood cultures in the matched analysis suggests that our propensity scores may not have fully captured patient severity of illness. In addition, we could not confirm the timing of blood culture obtainment in all cases, so we could not rule out some blood cultures being obtained for complications during hospitalization. Both these factors would bias the blood culture group towards longer lengths of stay. However, markers of disease severity and complications were only mildly higher in the blood culture group, e.g. 4% more ICU admissions and 5% more chest tube placements. Thus, the degree of bias from these sources is likely small. Moreover, our use of the Peto-Peto-Prentice test statistic to underweight late discharges limited the influence of prolonged lengths of stay. True prevalence of bacteremia may be inaccurate as not all patients were sampled and our analysis cannot account for false negative blood cultures due to antibiotic pre-treatment or limits of detection of bacteremia using current blood culture techniques. However, by repeating the Kaplan-Meier analysis excluding known bacteremia, we identified the maximum hypothetical benefit in LOS if bacteremia could be predicted with 100% accuracy. Our repeat analysis excluding pathogenic blood cultures matched controls with similar disease severity as the culture-negative patients, thereby decreasing risk of missed blood cultures. False negative blood cultures would by their very definition be unable to impact LOS as they would be interpreted by the practitioner in a similar fashion to true negatives. Hospital readmission could be one indirect method of identifying patients with false negative blood cultures, and no readmission among patients included in our study was due to bacteremia or sepsis. Additionally, because our study only included hospitalized patients, we excluded children who may have had blood cultures obtained in the ambulatory setting and were subsequently discharged, essentially resulting in a LOS of 0 days. Such patients would not typically be considered to have moderate or severe CAP if they were discharged, which makes blood culture obtainment in these patients more optional by most recommendations. Our assessment of vaccination status assumes a high prevalence of hospitalized children being completely vaccinated against pneumococcus and Hib. However, our method of assessment likely reflects clinicians’ perceptions of hospitalized patients’ vaccination status in the absence of refuting evidence.[24] Finally, blood culture obtainment may be related more to a general practice pattern of diagnostic testing and patient management which was unaccounted for in our propensity score. However, such confounding would only be present if the same person makes the decision to get blood cultures (and other testing) as the one who decides discharge, which is generally not the case for patients referred from ambulatory settings, particularly the ED.

## Conclusion

Obtaining blood cultures in children hospitalized with CAP rarely results in identification of a causative pathogen and is associated with increased LOS. Our findings highlight the need to further refine the role of obtaining blood cultures in children hospitalized with CAP.
